# Proteomics identification of novel fibrinogen-binding proteins of *Streptococcus suis* contributing to antiphagocytosis

**DOI:** 10.3389/fcimb.2015.00019

**Published:** 2015-03-04

**Authors:** Yaya Pian, Pingping Wang, Peng Liu, Yuling Zheng, Li Zhu, Hengliang Wang, Bin Xu, Yuan Yuan, Yongqiang Jiang

**Affiliations:** ^1^State Key Laboratory of Pathogen and Biosecurity, Academy of Military Medical Sciences, Beijing Institute of Microbiology and Epidemiology BeijingChina; ^2^National Center of Biomedical Analysis, Academy of Military Medical SciencesBeijing, China

**Keywords:** *Streptococcus suis* serotype 2, fibrinogen-binding proteins, interaction, antiphagocytosis, human blood

## Abstract

*Streptococcus suis* serotype 2 (SS2) induced sepsis and meningitis are often accompanied by bacteremia. However, the mechanism whereby it helps *S. suis* to evade PMN-mediated phagocytosis remain unclear. Because of the central roles of bacteria-human fibrinogen (hFg) interaction in innate immunity, here, a proteomics based Far-western blotting (PBFWB) was developed to identify the fibrinogen-binding surface proteins of *S. suis* (SsFBPs) on a large-scale. And then thirteen potential SsFBPs were identified by PBFWB and we selected seven potential surface proteins to further confirm their binding ability to hFg, of which the gene mutant strains of MRP displayed significantly decrease in binding to immobilized hFg. Additionally, the polyclonal antibodies against Enolase were found to significantly inhibit the binding of SS2 to hFg. Strikingly, MRP and Enolase were found to improve the antiphagocytic ability of SS2 to PMNs by interacting with hFg and enhance the survival of SS2 in human blood. Taken together, the PBFWB method provides useful clues to the bacteria-host interactions. These studies firstly disclose MRP and Enolase were involved in immune evasion of SS2 at least in part by binding to Fg, which make them potential targets for therapies for SS2 infection.

## Introduction

*Streptococcus suis* serotype 2 (SS2) infection is one of the major causes of septicemia and meningitis in pigs and humans (Wertheim et al., 2009; Huong et al., 2014), which are often accompanied by bacteremia (Tang et al., 2006; Wangkaew et al., 2006; Yu et al., 2006). To cause bacteremia, bacterial pathogens need to evade PMN-mediated innate immunity and maintain a high level in human blood. Although SS2 has become a public health concern due to the increasing number of human *S. suis* cases reported in the literature over the past 10 years (Huong et al., 2014), little is known about how *S. suis* resistant to PMN's phagocytosis and its survive in the host blood.

For the establishment of survive and dissemination in blood, bacteria must first interact with host proteins in blood followed by a variety strategies for avoiding host immunity. Fibrinogen (Fg) is a heterogeneous dimerica plasma glycoprotein protein composed of three pairs of non-identical peptide chains named Aα, Bβ, and γ. Fg also participates in the innate immune defense through interactions with leukocytes that support leukocyte activation events and delay apoptosis (Rubel et al., 2001). Numerous Fg-binding proteins (FBPs) in Gram-positives have been identified, including the Group A streptococci (GAS) M proteins (Carlsson et al., 2005; Courtney et al., 2006) and serum opacity factor (SOF) protein (Courtney et al., 2002); Group B streptococci (GBS) FbsA (Pierno et al., 2006); Group G streptococci (GGS) FOG (Johansson et al., 2004); staphylococcal Fg-binding microbial surface component recognizing adhesive matrix molecules(MSCRAMMs): ClfA, SdrG and FnbpA (Deivanayagam et al., 2002; Ponnuraj et al., 2003; Keane et al., 2007). Although many of these proteins employ distinct Fg-binding mechanisms (Rivera et al., 2007), the end result is to manipulate Fg' s biology to enhance the microbe's survival in the host. In many cases these FBPs act as virulence factors that involved in innate immunity such as GAS avoiding phagocytosis by M protein to inhibit the complement activation (Carlsson et al., 2005; Courtney et al., 2006), FOG induced the bacteria aggregation (Johansson et al., 2004), interference the recognization signal of phagocyte cells by Efb in *Staphylococcus aureus* (Ko et al., 2011), formation of a semi-flexible polymer-like network by interacting with FbsA in *Streptococcus agalactiae*, which becomes an efficient mask against phagocytic clearance (Pierno et al., 2006). Generally, FBPs were identified by Far-western blotting such as ClfA (Wann et al., 2000), FnbpA (Wann et al., 2000), SdrG (Davis et al., 2001), FAI (Talay et al., 1996) in Group C streptococci (GCS) and FbsA (Schubert et al., 2002). Moreover, the linear binding sequence were identified in FbsA (Schubert et al., 2002) and FAI (Talay et al., 1996), respectively, suggesting that a proteomics based Far-western blotting (PBFWB) could be developed to identify FBPs in pathogens on a large-scale.

It has been suggested that numerous surface proteins of pathogens are crucial for pathogenesis. *In silico* analysis of the genome of the Chinese SS2 reference strain 05ZYH33 revealed 33 putative cell-wall-anchored proteins containing LPXTG-or related motif (Wang et al., 2009). Thus, far, only an adhesin FBPs was reported to bind Fg (De Greeff et al., 2002). However, it remains to be determined whether or not these surface proteins play critical roles in the survival of *S. suis* in host blood.

In the present study, we developed a proteomics based Far-western blotting analysis to identify the novel fibrinogen-binding surface proteins of *S. suis* (SsFBPs) on a large-scale. Seven important potential SsFBPs were further compared their binding ability to hFg; of which, MRP (muramidase-released protein) and Enolase were identified as important FBPs in the high virulent SS2 strain 05ZYH33. Surprisingly, MRP and Enolase were found to mediate the antiphagocytosis of *S. suis* to PMNs by interacting with hFg. MRP is an important epidemic marker with unknown mechanism in pathogenesis. Enolase is a surface antigen of *S. suis*, however, the precise contribution of Enolase to bacterial virulence is unknown. These results may provide useful insights for understanding the pathogenesis of *S. suis*.

## Experimental procedure

### Bacterial strains and growth conditions

One SS2 strain 05ZYH33 (Chen et al., 2007), originally isolated from a dead STSS patient in an outbreak in Sichuan, China, 2005 was selected in this study. The *S. suis* strain was maintained on Columbia blood agar supplemented with 5% sheep blood and the inoculum was cultured in Todd-Hewitt broth (THB) at 37°C and harvested at stationary growth phase for experimentation. A total of 100 μg/ml spectinomycin (Spc) (Sigma), 5 μg/ml chloromycetin (Cm) and 8 μg/ml erythromycin (Em) was used for the *S. suis* transformants, and 50 μg/ml of ampicillin (Amp) (Sigma) was applied to screen *E. coli* transformants. The commercial pMD18-T vector (Takara) or pEASY-T1 (TransGen Biotech) was utilized to clone PCR fragments for direct sequencing of gene. *E. coli* DH5α cells were maintained in Luria–Bertani (LB) broth or agar medium at 37°C for recombinant plasmid amplification. Bacterial strains and plasmids used in this study are listed in Table 1.

**Table 1:** **Bacterial strains and plasmids used in this study**.

**Strain or plasmid**	**Description^a^ or sequence**	**Source, reference, PCR products**
*E. coli* DH5α	Host for cloning vector	In this lab
05ZYH33	Virulent Chinese *S. suis* serotype 2 isolate	In this lab
ΔMRP	Gene *mrp* knockout mutant strain; Cm^R^	This study
ΔFhb	Gene *fhb* knockout mutant strain; Cm^R^	Pian et al., 2012
ΔSsads	Gene *ssads* knockout mutant strain; Cm^R^	Liu et al., 2014
Δ1538	Gene *1538* knockout mutant strain; Cm^R^	This study
Δ1083	Gene *1083* knockout mutant strain; Cm^R^	This study
**Plasmids**	**Description**	
pMD18-T	TA cloning vector, lacZ, Amp^R^	TaKaRa
pEASY-T1	TA cloning vector, lacZ, Amp^R^	TransGen Biotech
pSET1	*S. suis*-*E. coli* shuttle vector, Cm^R^	Takamatsu et al., 2001
pSET4s	Gene replacement vector with MCS of pUC19, Spc^R^	Takamatsu et al., 2001
pSET4s:: *mrp*	The gene *mrp* knockout plasmid, Spc^R^ Cm^R^	This study
pSET4s:: *1538*	The gene *1538* knockout plasmid, Spc^R^ Cm^R^	This study
pSET4s:: *1083*	The gene *1083* knockout plasmid, Spc^R^ Cm^R^	This study
**Primers**	**Sequence^b^ (5′–3′)**	**PCR products**
*mrp* KOP1	CCC**AAGCTT**TGGAACGTGAGCGTTTGGCAAG	The *mrp* gene and its upstream flanking regions
*mrp* KOP2	CCTCGGAACCCATCGAATTACGACACACCAAGCAAAACG	
*mrp* KOP5	CATCAAGCTCTAGTTCGGTGTACTGGTGAGGCTTCATCTG	The *mrp* gene and its downstream flanking regions
mrp KOP6	CGC**GGATCC**CTCAAAGGATAGAGAGTTTGGAGCA	
*mrp*-F	GGAGCTGAAGTTGATGCCT	A fragment of *mrp* gene
*mrp*-R	GGTCGTTCTCCACAATTTCACG	
*1538*KOP1	GCA**GGATCC**AAAGAAGCCTTCACTGCAG	The *1538* gene and its upstream flanking regions
*1538*KOP2	CCTCGGAACCCATCGAATTAAGCTTGTGTTGCAATAACGC	
*1538*KOP5	CATCAAGCTCTAGTTCGGTGATACCGGTCAAGAAGC	The *1538* gene and its downstream flanking regions
*1538*KOP6	GCG**GAATTC**GTTATGAAGTCTTCGGCGC	
*1538*-F	GGACTTGATGAGTATAACCG	A fragment of *1538* gene
*1538*-R	GCCCGTTGTACCGTTTGTAT	
*1083*KOP1	GCA**GGATCC**GTGCAGTTCTTGTAGCTAAGACAGG	The *1083* gene and its upstream flanking regions
*1083*KOP2	CCTCGGAACCCATCGAATTAGATGCTTCTGAGCT	
*1083*KOP5	CATCAAGCTCTAGTTCGGTGGGTGTTGAATTGGCAG	The *1083* gene and its downstream flanking regions
*1083*KOP6	GCG**GAATTC**CACCGCTTGTAGGTTCC	
*1083*-F	TGTGTTCGGTATTGGTTTTGCC	A fragment of *1083* gene
*1083*-R	TGTACCTTCTGCTGATTGG	
CM-F	TAATTCGATGGGTTCCGAGG	Chloramphenicol resistant gene
CM-R	CACCGAACTAGAGCTTGATG	
SPC-F	GTGTTCGTGAATACATGTTATA	Spectinomycin resistant gene
SPC-R	GTTTTCTAAAATCTGATTACCA	

a Amp^R^, ampicillin resistant; Cm^R^, chloramphenicol resistant; Spc^r^, spectinomycin resistant; Em^R^, erythromycin resistant.

b The underlined sequences are the restriction sites.

### Ethics statement

The healthy donors who provide the serum and plasma in the 307 hospital in this study provided written informed consent in accordance with the Declaration of Helsinki. Approval was obtained from the medical ethics committee of the 307 hospital.

#### Identification of novel SsFBPs by proteomics-based far-western blotting (PBFWB)

The cell wall proteins (CWPs) used for proteome analysis was prepared as previous described (Geng et al., 2008). Briefly, bacterial pellets were resuspended in a separation buffer containing 30 mM Tris-HCl (pH 7.5), 3 mM MgCl_2_, 25% sucrose and mutanolysin (125 U/ml) and incubated for 1.5 h at 37°C. The protoplast fraction was separated by centrifugation at 12,000 rpm for 10 min at 4°C, and the supernatant containing CWPs was subject to the acetone-TCA precipitation. The CWPs were solubilized in a buffer containing 7 M Urea, 2 M Thiourea, 4% (w/v) CHAPS, 50 mM DTT and protease inhibitor cocktail. Protein concentrations were determined by the PlusOne 2-D Quant Kit (GE Healthcare).

The 2-DE experiment was carried out as previously described (Jing et al., 2008). Briefly, 18− mm immobilized pH gradient strips (pH 4-7; GE Healthcare Waukesha, WI, USA) were used in the isoelectric focusing (IEF) analysis, in which, 1 mg cell wall proteins resuspended in 350 μl rehydration buffer [7 M Urea, 2 M thiourea, 4% (w/v) CHAPS, 50 mM DTT] was loaded. For ligand blot analysis, the 2-DE gel was transferred to polyvinylidene difluoride (PVDF) membranes (Millipore), which were blocked with 5% w/v nonfat dry milk at 4°C for 12 h and incubated with 5 μg/ml hFg (Merck) for 1 h at room temperature, washed three times with Tris-buffered saline with Tween 20 (TBST) and incubated with HRP conjugated goat anti-hFg polyclone antibody (1:20000) (Abcam). The negative control group was incubated with TBST containing 1% BSA (-hFg). Positive spots were detected by enhanced chemi-luminescence western blotting detection reagents (Thermo Scientific, Waltham, MA, USA) and by exposing the membrane to X-ray film(Fuji photo film) at room temperature for 30–120 s.

### MALDI-TOF-MS to identification of positive spots

The positive protein spots in-Gel (the corresponding spots to Far-western blotting) digestion were carried out as described previously (Geng et al., 2008). Peptides from digested proteins were resolubilized in 2 μl of 0.5% trifluoroactic acid. The MALDI-TOF MS measurement was performed on a Bruker Reflex III MALDI-TOF MS (Bruker Daltonics, Germany) operating in reflectron mode with 20 kV accelerating voltage and 23 kV reflecting voltage. A saturated solution of R-cyano-4-hydroxy-cinnamic acid in 50% acetonitrile and 0.1% trifluoroactic acid was used as the matrix. One microliter of the matrix solution and sample solution at a ratio of 1:1 was applied onto the Score384 target well.

Database Searching was performed as described previously (Geng et al., 2008), to avoid inaccurate annotations derived from single genome or divergent protein sequences between strains, we established a local database including all the predicted protein sequences of three SS2 strains 98HAH33, 05ZYH33, and P1/7. Peptide mass fingerprints (PMFs) were searched by the program Mascot (Matrix Science Ltd.) licensed in-house against this local combined database, and the results were checked using Mascot with free access on the Internet (http://www.matrixscience.com) against NCBInr database. Monoisotopic masses were used to search the databases, allowing a peptide mass accuracy of 0.3 Da and one partial cleavage. Oxidation of methionine and carbamidomethyl modification of cysteine was considered. For unambiguous identification of proteins, more than 5 peptides must be matched for MALDI-TOF data.

#### Cloning, expression, and purification of recombinant potential SsFBPs

Seven potential surface SsFBPs were selected to be recombinant expressed, of which, six proteins were expressed the whole-length protein. MRP-N (N-terminus of MRP, a.a. 48–721) was expressed because the full length MRP protein was not easily water-soluble expressed. To express recombinant potential SsFBPs proteins in *E. coli*, genomic DNA isolated from *S. suis* strain 05ZYH33 was used as template for all PCRs using the oligonucleotide primers described in Supplemental Table S1. PCR products were digested and ligated into the PET-28a vector (GE Healthcare). The ligation mixture was transformed into *E. coli* BL-21 (Stratagene), grown on a LB agar plate containing 100 μg/ml kanamycin to select for transformants. Insertions were confirmed by DNA sequencing. Expression of fusion proteins was induced by adding 1 mM IPTG when cultures had grown to an A_600_ of 0.4–0.6. After the cell growth continued for another 4 h at 37°C, an aliquot of 1 ml was harvested. The induced proteins were purified from cultures growing at 30°C for 7 h by Ni-chelating chromatography (GE Healthcare) according to the manufacturer's recommendations.

#### Preparation of the anti-enolase sera and IgG

To generate polyclonal antibodies against Enolase, purified Enolase recombinant proteins were injected subcutaneously into two female rabbits as previously described (Geng et al., 2008). Serum from the immunized rabbits was collected. The preimmune IgG and the anti-Enolase IgG were purified from the preimmune serum and the immune serum, respectively, by affinity chromatography (Pierce, Rockford, IL, USA) according to the manufacturer's instructions (Pierce).

#### ELISA-type binding assay

To detect the binding of hFg to immobilized potential SsFBPs, 96-well immulon microtiter plates (Thermo Scientific) were coated overnight at 4°C with 100 μL of 5 μg/ml potential SsFBPs. After blocking the wells with 3% BSA in PBS, 100 μL of hFg (1–10 μg/ml, Merck) were added, and the plates were incubated for 1 h. Frequently, the plates were washed three times with PBST (Phosphate-buffered saline with 0.05% Tween-20) and incubated with HRP conjugated goat anti-hFg polyclone antibody (1:20000, Abcam). The negative control group was incubated with PBST containing 1% BSA, without hFg (0 μg/ml hFg). The binding was quantified after adding the substrate o-phenylenediamine dihydrochloride (Sigma) by measuring the resulting absorbance at 450 nm in a ThermoMax ELISA microplate reader (Molecular Devices).

#### Generation of the gene mutant of potential SsFBPs

DNA fragments corresponding to the upstream and downstream regions of the genes encoding potential SsFBPs were amplified using primers pairs described in Table 1. The Cm cassette was amplified from plasmid pSET1 with primers CM-F/CM-R. The primers pairs (Table 1) were designed to be fused as an intact fragment by overlap extension PCR. PCR amplicons were cloned into the temperature-sensitive *S. suis*-*E. coli* shuttle vector pSET4s, giving rise to the knockout vector pSET4s::*ssfbps*. The procedures for the selection of mutants by double cross-over were described previously (Takamatsu et al., 2001). The resulting mutant strain was verified by PCR using three pairs of primers (Table 1) and direct DNA sequencing analysis of the mutation sites using genomic DNA as template.

#### Binding of *S. suis* to immobilized fibrinogen

Binding of *S. suis* to immobilized fibrinogen was detected as previously described (Seo et al., 2012). Purified fibrinogen (100 μl of 1, 5, 10 μg/ml) or BSA (100 μl of 1, 5, 10 μg/ml) was immobilized in 96-well microtiter dishes by overnight incubation at 4°C and washed four times with PBS. Overnight cultures of the WT and the mutant strains were harvested by centrifugation and adjusted to almost equal original inoculum (concentration of ~10^5^ CFU/ml) in PBS. The wells pretreated with hFg/BSA were and then incubated with 100 μl of *S. suis* suspension for 30 min at 37°C. The wells were then washed four times with PBS to remove unbound bacteria, and then treated with 100 μl of trypsin (2.5 mg/ml) for 10 min at 37°C to release the attached bacteria. The number of bound bacteria was determined by plating serial dilutions of the recovered bacteria onto THB agar plates.

#### PMN killing assays

PMNs are the first line of defense against bacterial infections in human blood. To determine whether the interaction between SS2 and hFg has an impact on the antiphaocytosis ability of SS2, bacterial growth was measured in the presence of PMNs suspended in fresh plasma, fresh serum, or fresh serum supplemented with hFg. In PMN killing assay, PMNs were isolated from heparinized venous blood as described by Baltimore et al. (1977). Killing experiments of SS2 by PMNs were carried out as described previously (Pian et al., 2012). PMNs were infected with SS2 at MOI 1:15 (PMN: bacterium) in 50% non-immune human serum (or 50% human plasma, 50% serum supplemented with hFg) and centrifuged at 380 rpm for 5 min at 4°C. The plates were incubated at 37°C under 5% CO_2_for 60 min, samples were treated with 1% saponin and diluted, which were then plated onto blood agar. Colonies were counted and the percentage of surviving SS2 was calculated as follows: (CFU _PMN+_/CFU _PMN−_) × 100%. In antibodies blocking assay, the polyclonal antibodies against MRP-N or Enolase were preincubated with bacteria at 37°C for 15 min at room temperature before bacteria were added to PMNs.

#### Bactericidal assays by human blood

The bactericidal assays were used to compare the viability of WT and *ssfbps* mutant growth in human blood. Lancefield bactericidal assays were performed as described previously (Yamaguchi et al., 2006). Briefly, diluted cultures of WT, mutant (50 μl, ~10^4^ CFU/ml) were combined with fresh human blood (450 μl) and the mixtures were rotated at 37°C. At which time aliquots were incubated on ice for 20 min in a final concentration of 0.1% saponin to lyse eukaryotic cells. Viable cell counts were determined by plating diluted samples onto blood agar. The percent of live bacteria was subsequently calculated as (CFU on plate/CFU in original inoculum) × 100%. The data are presented as mean ± SD from three separate experiments.

#### Statistical analysis

Unless otherwise specified, all the data were expressed as means ± standard deviations. Differences between the wild-type strain and the isogenic mutant was analyzed using the unpaired two-tailed Student *t*-test. For all tests, a value of P < 0.05 was considered as the threshold for significance. All statistical tests were carried out using SPSS 15.0.

## Results

### The hFg enhances the survival of SS2 in PMN-killing assay

**Figure 1:**
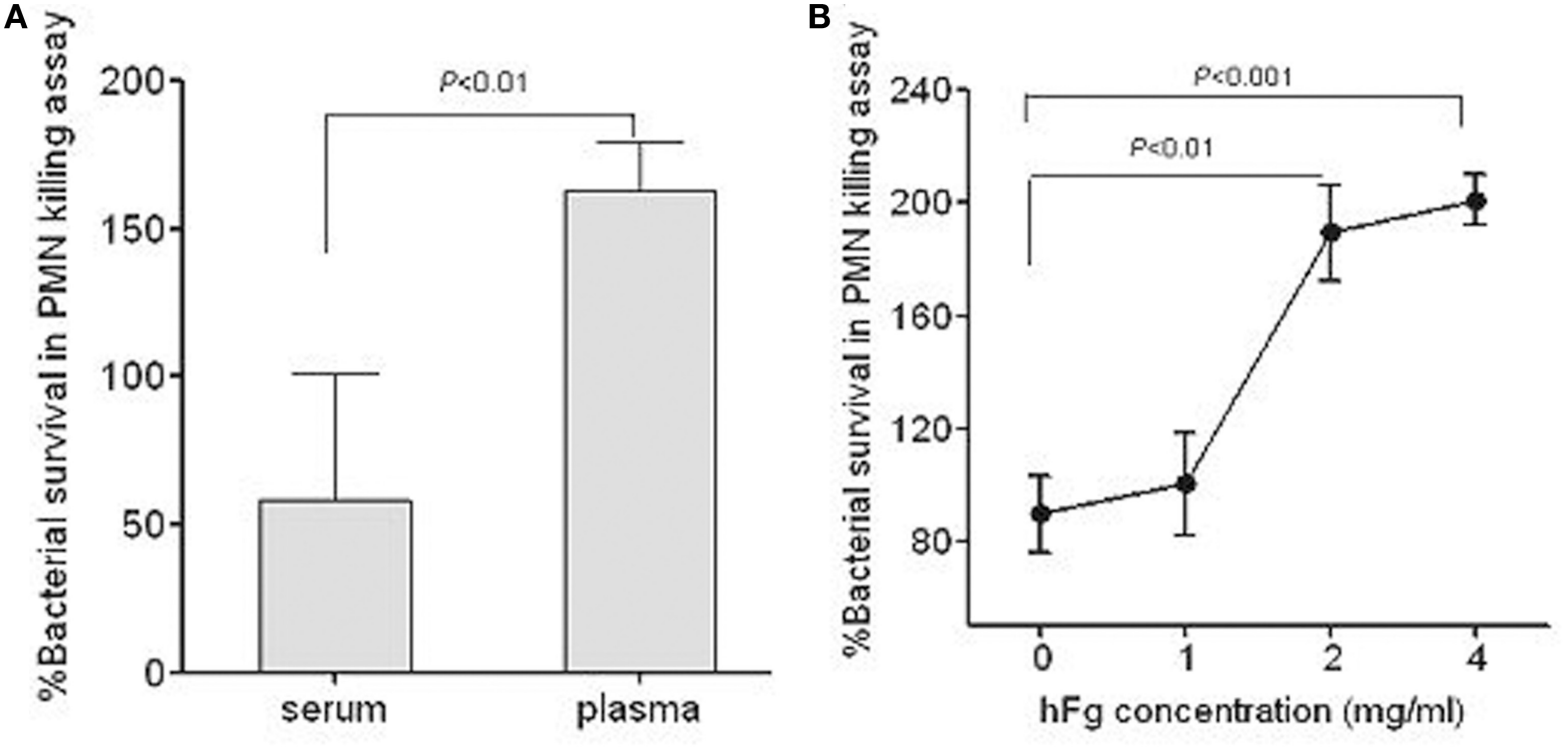
**Role of fibrinogen in resistance of *S. suis* to phagocytosis**. Viability of *S. suis* was enhanced in the presence of hFg, as determined by the PMN killing assay. The bacteria were co-incubated with PMNs at a MOI of 1:15 (PMN:bacterium) in 50% serum(S), 50% fresh plasma (P) or 50% serum supplemented with hFg 1, 2, or 4 mg/ml. At 60 min, PMNs were lysed by 1% saponin and bacteria were plated on solid agar medium. The percentage of viable bacteria was calculated as (CFU _PMN+_/CFU_PMN−_) × 100%. Significant differences were noted between the serum and the plasma **(A)**, or the serum (0 mg/ml hFg) and the serum supplemented with hFg at physiological concentrations (2–4 mg/ml) **(B)**. The serum and plasma isolated from the same healthy donor. Data are expressed as the mean ± SD of three independent experiments. hFg, human fibrinogen.

PMNs are the first line of defense against bacterial infections in human blood. To determine whether the interaction between SS2 and hFg has an impact on the survival of SS2 in PMNs killing assay, bacterial growth was measured in the presence of PMNs suspended in fresh plasma, fresh serum, or fresh serum supplemented with hFg, which is the main protein extracted when blood is clotted to produce serum. 05ZYH33 had superior growth when cultured with PMNs suspended in plasma as compared to serum (Figure 1A), with the addition of purified hFg at physiological concentrations (2–4 mg/ml) to the serum further enhancing growth (Figure 1B), suggesting that hFg is an important component that is required for SS2 to resist phagocytosis in human blood and potential contributions of FBPs to this resistance.

### Identification of novel SsFBPs by proteomics-based far-western blotting (PBFWB)

**Figure 2:**
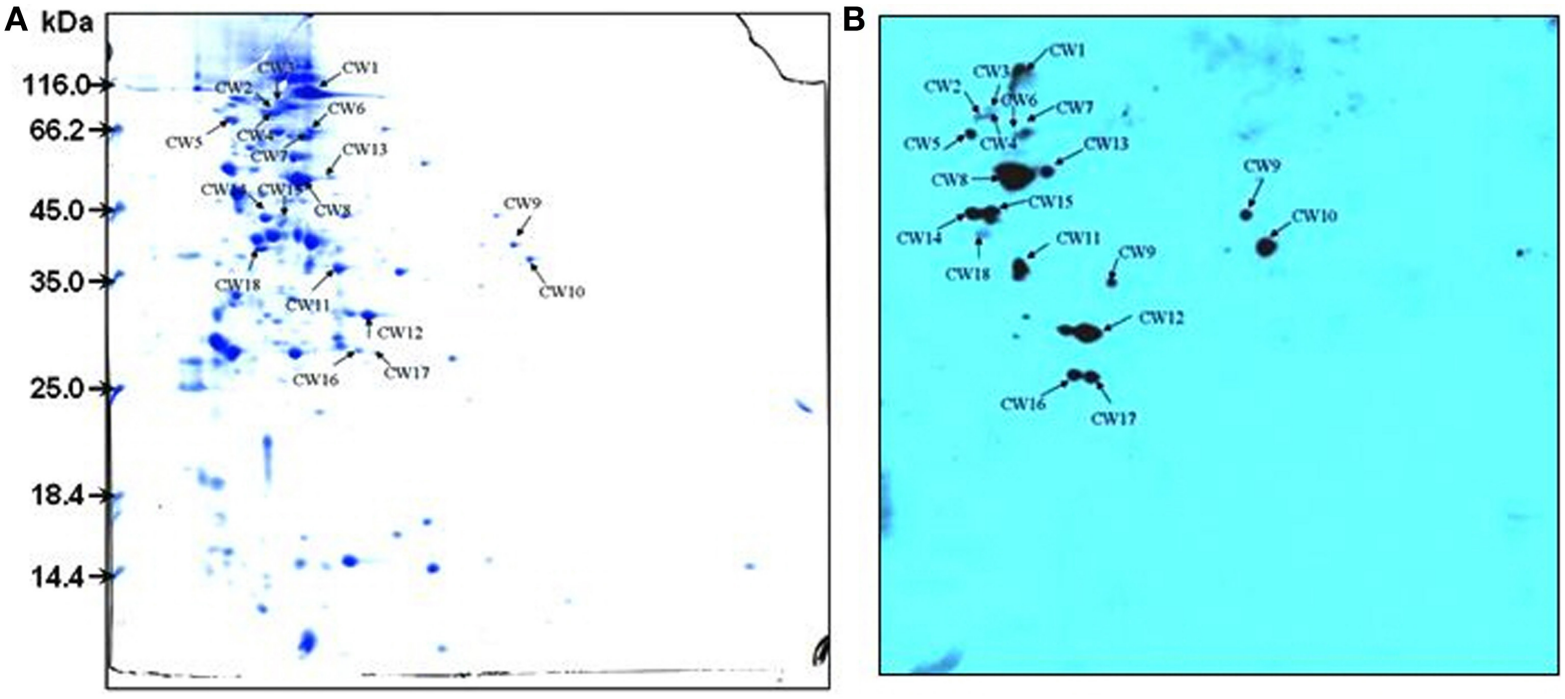
**2-DE profile (A) and Far-western blotting (B) identification of SsFBPs of *S. suis* CWPs**. The CWPs were separated in the first dimension (18 cm) by isoelectric focusing (IEF) in the pI range of 4–7 and by 12.5% SDS-PAGE in the second dimension. Arrows indicate potential SsFBPs recognized with HRP-anti human Fg polyclonal antibody. CWPs, cell wall proteins; SsFBPs, *S. suis*, fibrinogen-binding proteins.

To identify candidate SsFBPs contributing to the antiphagocytic activity of SS2, a proteomics-based Far-western blotting method was developed in the present study. Cells cultured in Todd Hewitt broth (THB) for 8 h before collection. Concentrated CWPs samples were analyzed by two-dimensional electrophoresis (2-DE) image analysis and Fg-binding positive spots were identified by Far-western blotting. Consistent with our previously study (Geng et al., 2008), more than 300 Coomassie-stained protein spots were detected on the 2-DE gel (Figure 2A) and 18 spots were detected by Far-western blotting (Figure 2B). No strong response spots were seen in the negative control (the transferred PVDF membrane was incubated in TBST containing 1% BSA without hFg). A total of 16 positive spots representing 13 proteins were identified by matrix-assisted laser desorption/ionization time-of-flight mass spectrometry (MALDI-TOF MS), as summarized in Table 2. Four proteins MRP, Fhb, Ssads and SSU05_1538 (HP1538) were predicted to be cell wall proteins based on the presence of the LPxTG motif typical of the membrane-anchored surface proteins in Gram-positive bacteria, of which MRP, factor H-binding protein (Fhb) and *S. suis* adenosine synthase (Ssads) have been reported as surface proteins for SS2 (Chen et al., 2010; Zhang et al., 2011; Liu et al., 2014). Two cytoplasmic enzymyes Enolase and glyceraldehyde-3-phosphate dehydrogenase (GAPDH) have also been documented as surface proteins involved in SS2 adherence (Wang and Lu, 2007; Feng et al., 2009). As an adhesin, Enolase also binds to fibronectin (Esgleas et al., 2008); indeed, most fibronectin-binding proteins also bind Fg in bacteria (Piroth et al., 2008; Burke et al., 2010). Three proteins SSU05_1868 (HP1868), SSU05_1869, and SSU05_1083 were putative ATP-binding cassette transporters, which are often transmembrane proteins in Gram-positive bacteria. Three cytoplasmic proteins SSU05_0252, SSU98_0330, and SSU05_0152 (annotated as glutamate dehydrogenase, fructose-bisphosphate aldolase and elongation factor G, respectively) could be due to contamination released by dead bacteria. These results provide a global view of hFg-binding proteins in SS2 and clues to the function of SsFBPs in SS2-induced pathogenesis.

**Table 2:** **Identification of the potential SsFBPs by MALDI-TOF MS**.

**Spot ID^a^**	**Score^b^**	**Locus^c^**	**GI**	**Product**	**Cellular location^g^**	**Sequence coverage^d^ (%)**	**Matched peptides^e^**
CW1	472	SSU05_0753	gi|146318407	MRP	Cell wall^h^	38	33
CW2	111	SSU05_0272	gi|146317928	Fhb	Cell wall^h^	26	13
CW3	148	SSU05_0272	gi|146317928	Fhb	Cell wall^h^	27	14
CW4	214	SSU05_1000	gi|146318654	Putative 5′-nucleotidase	Cell wall^h^	44	18
CW5	238	ssu05_1538	gi|146319192	Putative 5′-nucleotidase	Cell wall	40	29
CW6	285	SSU05_1868	gi|146319522	Peptide ABC transporter, peptide-binding protein	Non-Cytoplasmic	58	22
CW7	241	SSU05_1868	gi|146319522	Peptide ABC transporter, peptide-binding protein	Non-Cytoplasmic	63	23
CW8	295	SSU05_1503	gi|146319156	Enolase	Cytoplasmic^h^	60	22
CW9	241	SSU05_0155	gi|146317813	Glyceraldehyde-3-phosphate dehydrogenase	Cytoplasmic^h^	56	18
CW10	260	SSU05_0252	gi|146317908	gdhA; NADP-specific glutamate dehydrogenase	Cytoplasmic	48	24
CW11	361	SSU05_2086	gi|146319740	High-affinity zinc uptake system protein znuA precursor	Non-Cytoplasmic	42	13
CW12	257	SSU98_0330	gi|146320177	Fructose-bisphosphate aldolase	Cytoplasmic	39	10
CW13	521	SSU05_0152	gi|146317810	Elongation factor G	Cytoplasmic	42	27
CW14	482	SSU05_1548	gi|146319202	Hypothetical protein	Non-Cytoplasmic	33	13
CW15	696	SSU05_0152	gi|146317810	fusA; elongation factor EF-G	Cytoplasmic	43	26
CW16	UI^f^	UI^f^					
CW17	UI^f^	UI^f^					
CW18	327	SSU05_1083	gi|146318737	ABC transporter periplasmic protein	Non-Cytoplasmic	40	11

a Spots referring to Figure 2.

b Total protein score based on combined mass and mass/mass spectra.

c Three different kinds of loci in the genome annotations of three S. suis strains (98HAH33, 05ZYH33, and P1/7) are used. Proteins with high homology were assigned with a locus in the genome annotation of the 98HAH33 strain.

d Percentage of the identified sequence to the complete sequence of the known protein.

e All the spots had high-probability results by MASCOT search, and there is at least one peptide analyzed by MS/MS in each spot.

f Unidentified.

g Cellular location was predicted by PSORTb (http://www.psort.org/psortb/index.html).

h Which have been reported as surface proteins.

### Confirmation of the interaction of hFg with SsFBPs

**Figure 3:**
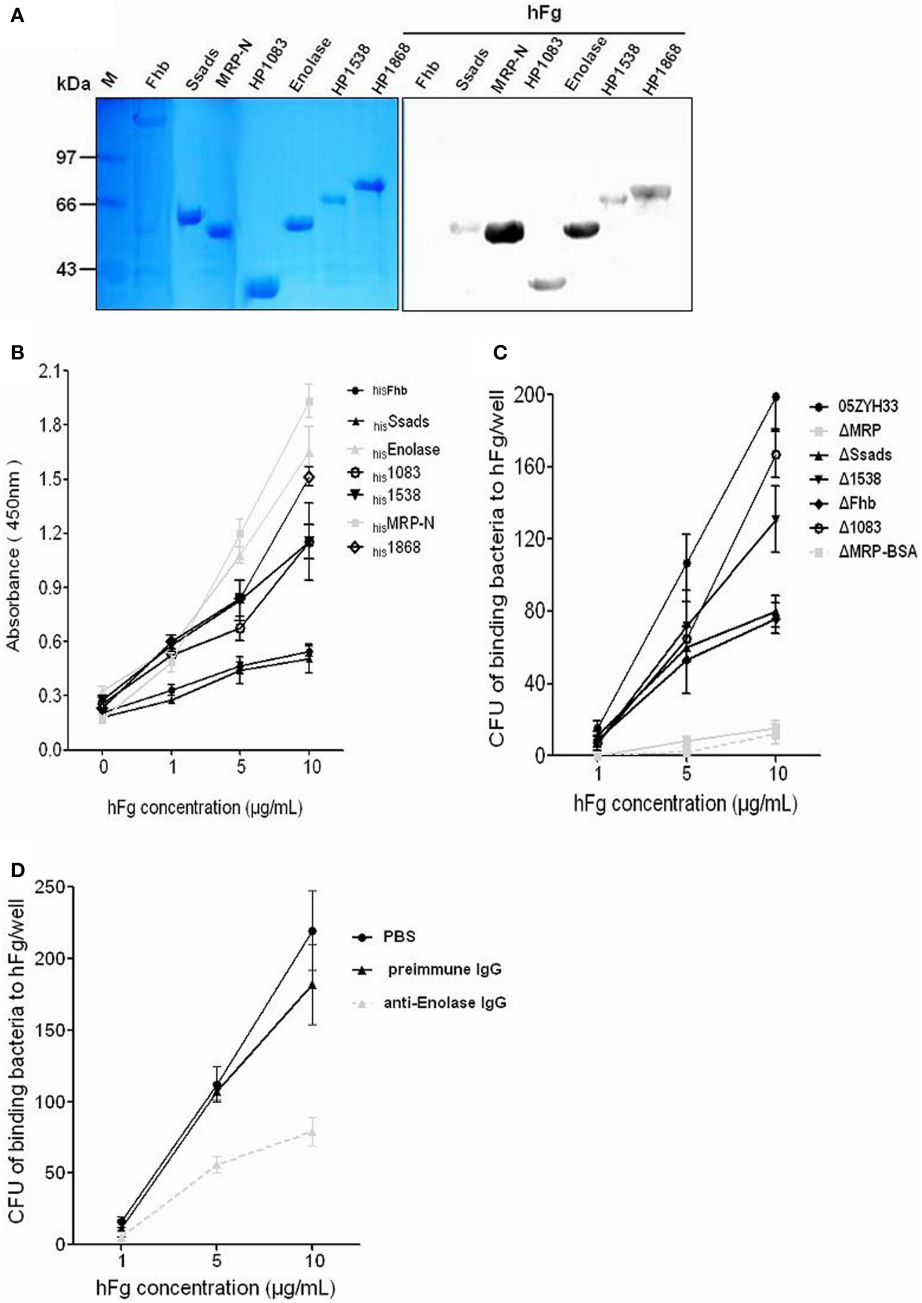
**Identification the binding of fibrinogen to FBPs of *S. suis*. (A)** The hFg-binding capacity of recombinant SsFBPs was assessed by Far-western blotting; _his_Fhb, _his_Ssads, _his_MRP-N, _his_1083, _his_enolase, _his_1538, and _his_1868 were separated by SDS-PAGE and then transferred to PVDF and incubated with hFg (5 μg/ml). Bound hFg was detected with HRP conjugated anti-hFg antibody. **(B)** Microtiter wells were coated with purified recombinant putative SsFBPs and reacted with the indicated concentrations of hFg. The negative control was the incubation of immobilized recombinant SsFBPs with PBST containing 1% BSA, but without hFg (0 μg/ml). Bound hFg was detected with HRP conjugated anti-hFg antibody. Data are expressed as the mean ± SD of three independent experiments. **(C)** Binding of the WT and the *ssfbp* mutants SS2 to immobilized hFg. SS2 strains WT and the *ssfbp* mutants (~1 × 10^4^ CFU per well) were incubated with wells coated with hFg or BSA (1, 5, 10 μg/ml per well). Data are expressed as the mean ± SD of three independent experiments. **(D)** Binding of WT SS2 to immobilized hFg after blocked with specific antibodies. *SS2* strains WT (~1 × 10^4^ CFU per well) that had been pretreated with preimmune IgG or the anti-Enolase IgG were incubated with wells coated with hFg (1, 5, 10 μg/ml per well). Values represent the Data are expressed as the mean ± SD of three independent experiments. WT, wild-type (05ZYH33); ΔMRP, the isogenic mutant strain of *mrp*; Δ1538, the isogenic mutant strain of *1538*; Δ1083, the isogenic mutant strain of *1083*; ΔFhb, the isogenic mutant strain of *fhb*; ΔSsads, the isogenic mutant strain of *ssads*. ΔMRP-BSA, the binding of ΔMRP strains to BSA (control to hFg).

We selected the above seven potential surface SsFBPs for recombinant expression and further compare their ability to binding to hFg by Far-western blotting or ELISA-type binding assay. A strong response to hFg was detected for of the N-terminus of MRP (_his_MRP-N, a.a. 48–721) and _his_Enolase by Far-western blotting (Figure 3A). The ability of _his_Ssads, _his_Fhb, _his_1083, _his_1538, _his_1868, _his_MRP-N, and _his_Enolase to bind hFg was further evaluated by ELISA-type binding assay, _his_MRP-N,_his_Enolase,_his_1868, _his_1538, and_his_1083 showed concentration-dependent binding to Fg (Figure 3B); with similar results to Far-western blotting, _his_MRP-N and_his_ Enolase displayed strong binding to hFg. Obviously, OD_450_ of MRP and Enolase group was 4-fold and 3-fold than Fhb at 10 μg/ml hFg, suggesting that MRP and Enolase were the important SsFBPs; but 1868, 1083, and 1538 also bound fibrinogen to a significant degree. However, the recombinant SsFBPs couldn't reflect the genuine abundance of proteins on bacteria. Considering most of the gene encoding these SsFBPs don't lie in close proximity of phage-associated genes with their independent promoters, it's therefore to construct the gene knockout mutants of these SsFBPs and evaluate the binding ability of WT and these *ssfbps* mutants to immobilized hFg. Interestingly, the results indicate the mutant ΔMRP, in which MRP was inactivated, bound dramatically less fibrinogen than the parent strain (Figure 3C). Enolase is a functionally important enzyme in both prokaryotic and eukaryotic organisms (Gerlt et al., 2012) and its gene can't be knock out, the specific antibody-blocking assay was used in this study. The results showed that the polyclone antibody against the Enolase significantly decreased the adhesion of 05ZYH33 to hFg, respectively, while the preimmue IgG, as control, almost had no effect on these adhesion (Figure 3D), which is consistent with the result of ELISA-binding assay. The protein 1868 was not further studied for it's difficult to acquire its specific antibody and its mutant strain. These results suggest that MRP and Enolase are the major FBPs on SS2, they would possibly play some roles in *SS2*-induced pathogenesis.

### MRP and enolase increase antiphagocytic activity of SS2 to PMNs by interacting with hFg

**Figure 4:**
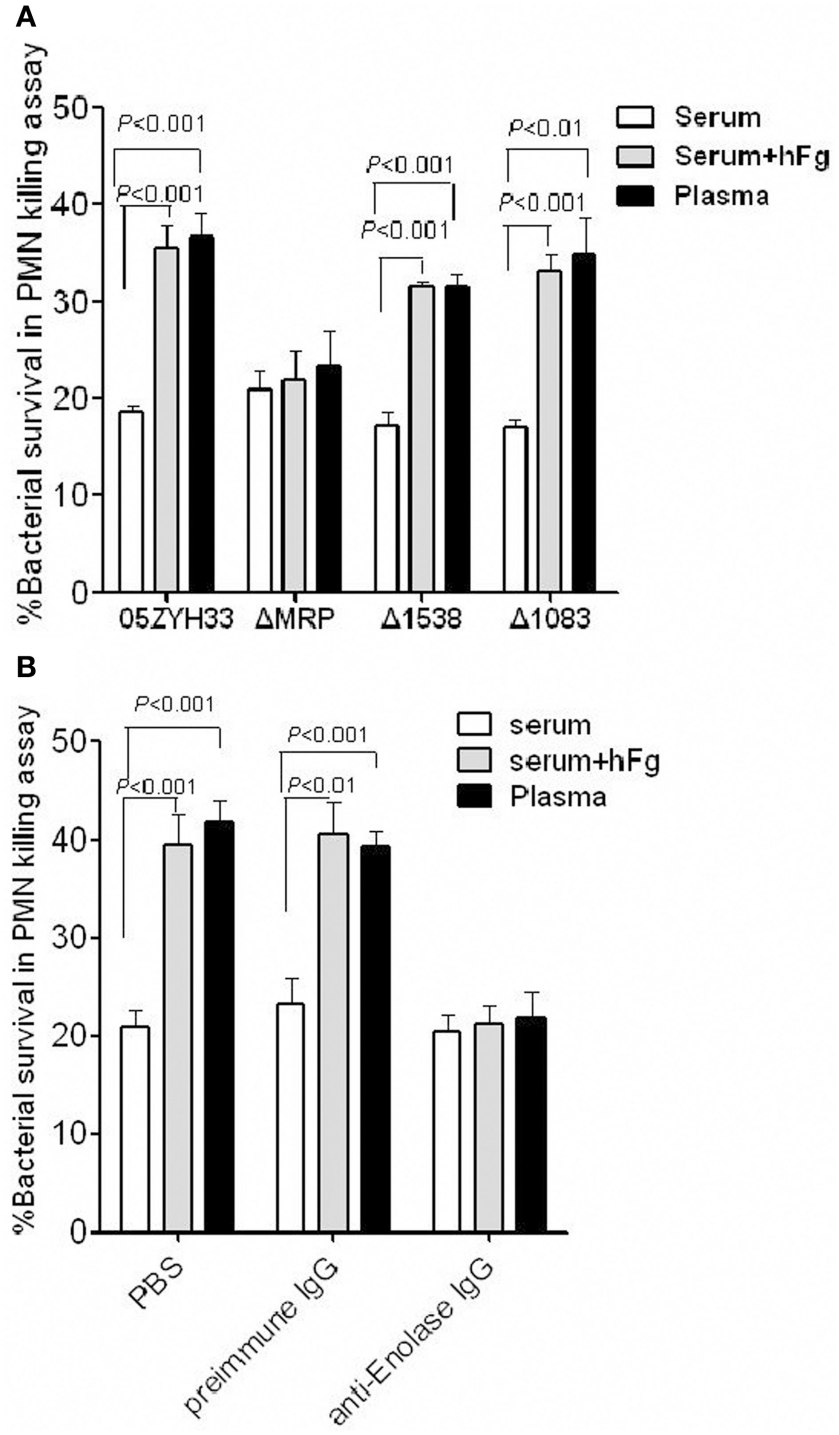
**MRP and Enolase improve the antiphagocytic ability of SS2 by interacting with hFg. (A)** The MRP-hFg interaction enhanced the survival of SS2 in PMN killing assays. WT, ΔMRP, Δ1538, and Δ1083 strains were co-incubated with PMNs at a MOI of 1:15 (PMN:bacterium) in 50% serum without or with hFg (2 mg/ml) for 60 min. The mutant ΔMRP significantly reduced viability compared to WT in both plasma and serum containing hFg at physiological concentrations (2 mg/ml), but not in serum alone. Specific antibodies against Enolase **(B)** significantly decrease the viability of SS2 in plasma or serum sulpplemented with hFg, but not in serum alone. Bacteria that pretreated with preimmune IgG (control), or anti-Enolase IgG were co-incubated with PMNs at a MOI of 1:15 (PMN:bacterium) in 50% serum without or with hFg (2 mg/ml) for 60 min. The percentage of viable bacteria was calculated as (CFU _PMN+_/CFU_PMN−_) × 100%. Data are expressed as the mean ± SD of three independent experiments. WT, wild-type (05ZYH33); ΔMRP, the isogenic mutant strain of *mrp*; Δ1538, the isogenic mutant strain of *1538*; Δ1083, the isogenic mutant strain of *1083*.

To determine whether the SsFBPs-hFg interactions have impacts on the antiphaocytosis ability of SS2, bacterial growth was measured in the presence of PMNs suspended in fresh plasma, fresh serum, or fresh serum supplemented with hFg. The data indicated that the *mrp* mutant ΔMRP, not the 1083 and 1538 mutant, was found to be more killed than the WT 05ZYH33 in plasma or serum supplemented with hFg oposonized PMNs, but not in serum alone (Figure 4A). Additionally, in a specific antibody blocking assay, comparing to preimmune serum, the antiserum against the Enolase (Figure 4B) decreased the bacteria survival in plasma or serum supplemented with hFg at physiological concentrations (2 mg/ml), but had no effect in serum alone. These results indicate that, among SsFBPs, MRP and Enolase are responsible for improving antiphagocytic ability of *S. suis* by interacting with hFg. Fhb and Ssads were reported as FH-binding protein and adenosine synthase of SS2 in our previous study, respectively; they also play important roles in immune evasion in human blood, therefore not be studied in the present study.

### MRP and enolase enhance the survival of SS2 in human blood

**Figure 5:**
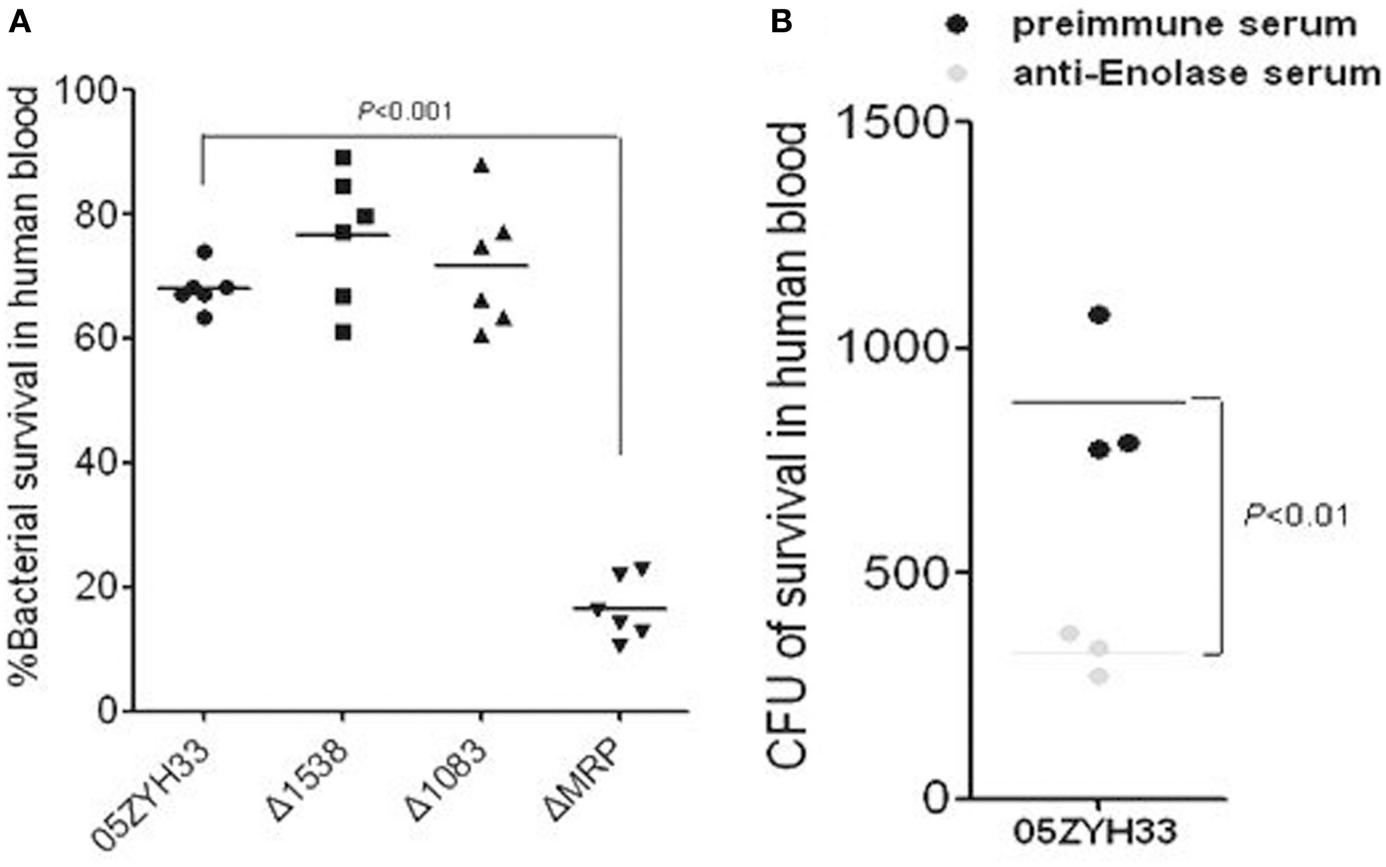
**MRP and Enolase contribute to the survival of *SS2* in human blood. (A)** Viability of the *ssfbp* mutant strains in whole human blood. Bacteria (50 μl, at ~2 × 10^4^ CFU/ml) were added to heparinized whole blood (450 μl) and then gently mixed for 60 min at 37°C. The bacterial CFU in human blood was determined by THB plates. Each symbol represents the percentage of live bacteria isolated from individual human blood. The percentage of live bacteria was subsequently calculated as (CFU on plate/CFU in original inoculum) × 100%. The horizontal lines indicate the mean for each group. Antibodies against Enolase **(B)** decrease the viability of SS2 in human blood. Bacteria were pretreated with preimmune sera (control), or anti-Enolase sera from rabbit (1:5 dilution). Each symbol represents the CFU/ml isolated from individual human blood. Horizontal lines indicate the mean for each group. Significant differences were found between preimmune serum and anti-Enolase serum blocking.

PMNs are the major phagocytes with defense against bacteria in host blood; the SsFBPs-hFg interaction improving the antiphagocytic ability of SS2 to PMNs suggest that these SsFBPs possibly contribute to the survival of SS2 in host blood. We performed bactericidal assays to test for the growth of WT and *ssfbp*-mutant strain or the strain with specific antibody-blocking in healthy non-immune human blood from different donors. As expected, it's ΔMRP, not Δ1538 and Δ1083, reduced the viability compared to WT (Figure 5A). Additionally, in a specific antibody blocking assay, the polyclone antibody against Enolase decrease the WT survival in human blood (Figure 5B), suggesting that MRP and Enolase play important roles in immune evasion of SS2 in host blood.

## Discussion

*Streptococcus suis* is an emerging pathogen to leading cause of skin wounds, blood-stream, brain (Segura, 2009). To survive in blood, Gram-positive pathogenic bacteria must escape a variety of innate immune mechanisms, such as complement-mediated phagocytosis. However, the mechanism of *S. suis* survival in human blood is largely unclear and few virulence factors were found to be involved in escaping innate immunity in host blood for SS2 such as Fhb and Ssads in our previous study (Pian et al., 2012; Liu et al., 2014).

Some surface proteins have been reported to bind to Fg in GAS and GBS that are involved in bacteria pathogenesis (Courtney et al., 2006; Pierno et al., 2006; Seo et al., 2012). Our results now demonstrate that hFg promotes the survival ability of SS2 in PMN-killing assay. However, the interaction between SS2 and hFg remained unclear, which may be mediated the surface proteins of SS2. In this work, we identified potential SsFBPs with proteomics based far-western blot method (PBFWB). Thirteen putative Ssfbps were identified, among those proteins, there are four proteins were predicted with a cell wall attachment consensus motif LPxTG; of which, three proteins (MRP, Fhb and Ssads) and two cytoplasmic enzymes (Enolase and GAPDH) have been reported as surface proteins for SS2 in previous studies (Wang and Lu, 2007; Feng et al., 2009; Chen et al., 2010; Zhang et al., 2011; Liu et al., 2014). The known fibrinogen-binding proteins of *S. suis*, FBPs (De Greeff et al., 2002), was not found by PBFWB, which might be due to its low abundance in the cell wall. The above five surface proteins and two putative ABC transporters (SSU05_1083 and SSU05_1868) were further compared their binding abilities to hFg. Interestingly, two proteins Enolase and MRP displayed strongly binding ability to hFg and proved to be the major SsFBPs on SS2. Moreover, among these proteins, only the isogenic mutant strain of *mrp* displayed decreased survival ability compared to the WT strain in the PMN killing assay in the presence of hFg; and the anti-enolase antibody decreased the 05ZYH33 survival in the presence of hFg in the PMN killing assay (Figure 4).

The muramidase-released protein (MRP), a 136-kDa protein, apart from being associated with the bacterial cell wall of *S. suis* with LPxTG motif, MRP is also released from the surface and detected in culture supernatants (Vecht et al., 1991), which is often reported as good markers of infection in Europe and Asia in epidemiology (Vecht et al., 1996; Yang et al., 2006; Rehm et al., 2007) but with low similarity to other function-known proteins by amino acid sequence analysis. Moreover, the contribution of MRP to bacterial virulence remained controversy because this protein was reported as not a critical virulence factor in an intranasally infection model in Anthony piglets (Smith et al., 1996). Here, our results now revealed that MRP-hFg interaction promotes the antiphagocytosis of SS2 to neutrophils and then enhances the survival of SS2 in human blood; this process is likely to be important for the pathogenesis of SS2 but is independent of the inhibition of the complement deposition on SS2 (data not shown). However, M proteins were the important FBPs and virulence factors for GAS, which inhibit the complement deposition on bacteria surface by binding of hFg to the surface M proteins (Carlsson et al., 2005; Courtney et al., 2006). Considering the antiphagocytosis roles of MRP in human blood, the contribution of MRP to the virulence in 05ZYH33 was suggested to be verified in an i.v. infection model in piglets.

Similar to GAPDH, enolase is both a cytosolic enzyme of the glycolysis pathway and a surface adhesion (Feng et al., 2009) involved in their interactions with plasmin(ogen) (Pancholi and Fischetti, 1998; Lu et al., 2012) and fibronectin (Esgleas et al., 2008). However, the role of Enolase in immune evasion remained unclear until now. In the present study, an intriguing observation was that enolase contribute to antiphagocytosis of SS2 by interacting with hFg. Moreover, the antibody against Enolase could decrease the survival of SS2 in human blood. Consisting with that, Enolase was reported as a surface antigen with significant protection against serotype 2 and 7 intra-peritoneal challenges in BALB/c mice (Zhang et al., 2009).

In summary, herein, novel FBPs of SS2 were identified on large scale by combining proteomic and immune blot; of which, two SsFBPs (MRP and Enolase) were identified as important FBPs of SS2. More interesting, MRP and Enolase contribute to antiphagocytic ability of SS2 by interacting with hFg. MRP is an important infection marker in most Chinese and Europe clinical isolates; Enolase is a conserve surface antigen among *S. suis* strains, the polyclone antibodies against them could decrease the survival of SS2 in human blood, it is therefore to be promising candidates for novel therapies for targeting SS2-induced sepsis.

### Conflict of interest statement

The authors declare that the research was conducted in the absence of any commercial or financial relationships that could be construed as a potential conflict of interest.
