# Supplementary material for: Proteomics identification of novel fibrinogen-binding proteins of Streptococcus suis contributing to antiphagocytosis
Source: Front Cell Infect Microbiol. 2015 Mar 4;5:19. doi: 10.3389/fcimb.2015.00019 (PMC4349166; doi:10.3389/fcimb.2015.00019)
Supplement: Supplementary file 1 [file Table1.DOC]

**Supplemental information**

**Table S1. Primers, Vectors, and Cloning Sites Used in SsFBPs recombinant Expression**

| protein | forward primer (5′- 3′) | reverse primer (5′- 3′) | vector/cloning sites |
| --- | --- | --- | --- |
| MRP-N  Fhb  Ssads  Enolase  HP1538  HP1083  HP1868 | GCGAATTCGAACAGGTAACATCAGAATCA  GTTGGATCCGAATCGCTAGAAC  CGGGATCCATGAACCAAGTCGGCTATGATGC  GGATCCTTGTCAATTATTACTGATGTTTAC  GCGGATCCAAAAAGAATATTCGGTTGAAAAG  GCGAATTCAACAAGAAACTTGTTGGACTG  GCGAATTCAAAAAGACAACGAAACTTTTTGC | ATCTCGAGGTCCAAACCTGAGTAGCGATATA  TATCTCGAGACTTGCTTCGCCTGTAT  CCGCTCGAGGACTCCTGCTTTTGTAGTTTTTG  AAGCTTTTATTTTTTCAAGTTGTAGAATGAGTTCAAGCC  ATCTCGAGCTACTCCCCTTCCTTACGTCTCA  ATCTCGAGAGGTTTTTCAGGAACTTCTAC  ATCTCGAGTTCTGCCACTACACCCTTATC | pET28a(+)/EcoRI, XhoI  pET28a(+)/BamHI, XhoI  pET28a(+)/BamHI, XhoI  pET28a(+)/BamHI,HindIII  pET28a(+)/BamHI, XhoI  pET28a(+)/EcoRI, XhoI  pET28a(+)/EcoRI, XhoI |
